# Unwanted Exacerbation of the Immune Response in Neurodegenerative Disease: A Time to Review the Impact

**DOI:** 10.3389/fncel.2021.749595

**Published:** 2021-10-22

**Authors:** Amanda de Oliveira Ferreira Leite, João Bento Torres Neto, Renata Rodrigues dos Reis, Luciane Lobato Sobral, Aline Cristine Passos de Souza, Nonata Trévia, Roseane Borner de Oliveira, Nara Alves de Almeida Lins, Daniel Guerreiro Diniz, José Antonio Picanço Diniz, Pedro Fernando da Costa Vasconcelos, Daniel Clive Anthony, Dora Brites, Cristovam Wanderley Picanço Diniz

**Affiliations:** ^1^Laboratório de Investigações em Neurodegeneração e Infecção, Hospital Universitário João de Barros Barreto, Instituto de Ciências Biológicas, Universidade Federal do Pará, Belém, Brazil; ^2^Laboratório de Microscopia Eletrônica, Instituto Evandro Chagas, Belém, Brazil; ^3^Seção de Arbovirologia e Febres Hemorrágicas, Instituto Evandro Chagas, Ananindeua, Brazil; ^4^Department of Pharmacology, University of Oxford, Oxford, United Kingdom; ^5^Research Institute for Medicines (iMed.ULisboa), Faculty of Pharmacy, Universidade de Lisboa, Lisbon, Portugal; ^6^Department of Pharmaceutical Sciences and Medicines, Faculty of Pharmacy, Universidade de Lisboa, Lisbon, Portugal

**Keywords:** mouse prion disease, virus infection, exacerbated inflammatory response, prion-like neurodegenerative diseases, exercise, sedentary lifestyle, microglia hyperactivation, GFAP astrocytes reactivity

## Abstract

The COVID-19 pandemic imposed a series of behavioral changes that resulted in increased social isolation and a more sedentary life for many across all age groups, but, above all, for the elderly population who are the most vulnerable to infections and chronic neurodegenerative diseases. Systemic inflammatory responses are known to accelerate neurodegenerative disease progression, which leads to permanent damage, loss of brain function, and the loss of autonomy for many aged people. During the COVID-19 pandemic, a spectrum of inflammatory responses was generated in affected individuals, and it is expected that the elderly patients with chronic neurodegenerative diseases who survived SARSCoV-2 infection, it will be found, sooner or later, that there is a worsening of their neurodegenerative conditions. Using mouse prion disease as a model for chronic neurodegeneration, we review the effects of social isolation, sedentary living, and viral infection on the disease progression with a focus on sickness behavior and on the responses of microglia and astrocytes. Focusing on aging, we discuss the cellular and molecular mechanisms related to immunosenescence in chronic neurodegenerative diseases and how infections may accelerate their progression.

## Introduction

The ongoing viral pandemic has imposed behavioral changes resulting in increased social isolation and a more sedentary life, which has affected all age groups (Schwabenland et al., 2021; Yang et al., 2021). However, social isolation during the COVID-19 pandemic especially affected the elderly population with comorbidities, who were already exhibiting mild or moderate cognitive deficits and senile cognitive decline associated with neurodegenerative diseases (Tangalos and Petersen, 2018; Juan and Adlard, 2019; Noguchi et al., 2021).

Older adults are more vulnerable to infectious diseases (Clark et al., 2020; Cunha et al., 2020) due to immune system dysregulation (Müller et al., 2019), together with cellular and signaling pathway impairments, which contribute to cell cycle arrest (Calcinotto et al., 2019), oxidative stress (Liguori et al., 2018), mitochondrial dynamic abnormalities (Kudryavtseva et al., 2016), autophagic disruption (Wong et al., 2020), immunosenescence (Fülöp et al., 2016; Pawelec, 2018), and neuroinflammation (Ransohoff, 2016). Dysregulation of these processes is known to be associated with the pathogenesis of neurodegenerative diseases (Brites, 2015; Schmeer et al., 2019; Wissler Gerdes et al., 2020). During the COVID-19 pandemic, these vulnerabilities have led to an increase in mortality rate that reached 1.4–15% in people in the age group between 65 and 85 years old, as compared with a much lower rate of 0.01–0.4% in the age group from 25 to 55 years (Levin et al., 2020). A meta-analysis of the infection-fatality rate has been estimated to be 0.53–0.82% (Meyerowitz-Katz and Merone, 2020). For those responsible for implementing the COVID-19 health policy, it is now clear that COVID-19 pathology extends well beyond lung pathology (Pannone et al., 2021) as is there now evidence of kidney damage (Gabarre et al., 2020; Hassanein et al., 2020; Ronco et al., 2020), pathological sequelae in the hepatobiliary, gastrointestinal, pancreatic (Jothimani et al., 2020; Lee et al., 2020; Patel et al., 2020), reproductive (He et al., 2021), cardiovascular (Bansal, 2020; Spuntarelli et al., 2020), and central nervous (Fiani et al., 2020; Nagu et al., 2021) systems. As a consequence, the potential for the interaction between the activated systemic immune system and neurodegenerative disease pathology is increased, and the mechanisms are likely to be more complex than previously envisaged.

The decline in physical activity imposed by restriction of outdoor activities and sedentary behaviors (Stockwell et al., 2021) is known to exacerbate chronic illnesses directly and has led to an increase in stress, anxiety, and depression that is also known to have an impact on comorbidities. For example, studies have highlighted that cardiovascular and cerebrovascular dysfunctions or kidney damage (Lee A. C. et al., 2021), metabolic disorders (Kullmann et al., 2016; Dye et al., 2017; Li et al., 2017; Bailly et al., 2021), motor impairments, and other chronic illnesses are aggravated by an increased sedentary life (Araújo et al., 2021; Awogbindin et al., 2021; de Boer et al., 2021; Engels et al., 2021; Salman et al., 2021), and thus these individuals are likely to require more medical attention and continued monitoring for potential long-term sequelae.

It is already known that SARS-CoV-2 binds to the receptor for angiotensin-converting enzyme 2 (ACE2) (Hoffmann et al., 2020; Zhang et al., 2020; Zhou et al., 2020), which is most prominently expressed by epithelial and endothelial cells, and, to a lesser extent, by neurons and glial cells (McQuaid et al., 2021; von Bohlen Und Halbach, 2021). The presence of SARS-CoV-2 in droplets in the air enters the upper respiratory tract, infecting the nasal and pharyngeal epithelia and the bronchial and alveolar epithelium (Bourgonje et al., 2020). In symptomatic patients, nasal swabs have shown higher viral loads than throat swabs (Zhou et al., 2020) owing to the high expression of ACE2 in the nasal epithelial cells (Sungnak et al., 2020). The viral protein Spike interacts with the ACE2 receptor in several different tissues, such as the central nervous system, where it increases angiotensin II and activates nicotinamide dinucleotide phosphate oxidase2 (NOX2) enzyme with the subsequent release of reactive oxygen species (ROS) and inflammatory mediators (Sindona et al., 2021).

Patients with SARS-CoV-2 show elevated levels of pro-inflammatory cytokines mediated by the dysregulation of the nuclear factor kappa B (NF-κB) signaling pathway (Hammoudeh et al., 2021; Su et al., 2021) and downstream enhanced expression of pro-inflammatory genes that translate into increased neuroinflammation (Liu et al., 2017). Although, some reports have addressed the potential long-term effects of chronic mild neuroinflammation in neurodegenerative diseases and the acceleration of progression rate (Alonso-Lana et al., 2020; Dewanjee et al., 2021), the persistence of neuroinflammatory events induced by SARS-CoV-2 on a background of neuropsychiatric and neurological sequelae (Carod-Artal, 2020; Dinakaran et al., 2020; Troyer et al., 2020; Wang et al., 2020; Yachou et al., 2020; Swain et al., 2021) have the potential to aggravate the pathophysiological aspects in the survivors (Perry, 2010; Holmes et al., 2011; Amor et al., 2014; Alam et al., 2017; Idrees and Kumar, 2021; Marques Zilli et al., 2021; Too et al., 2021).

Thus, we considered it to be of interest to review the potential consequences of the effects of the COVID-related inflammatory response on the immune responses linked to chronic neurodegeneration, associated with central or peripheral virus infections. To that end, we here revisited the influences of social isolation, sedentary life, and central or peripheral infections on mouse prion disease progression, as a proxy for the exacerbated immune response of prion-like chronic neurodegenerative diseases (Fernández-Borges et al., 2015; Armstrong, 2020; Goedert, 2020; Hosseini et al., 2021) under similar conditions.

### Experimental Mouse Prion Disease and Prion-Like Chronic Neurodegenerative Diseases

From a neuropathological point of view, several parallels have been established between prion diseases (Orge et al., 2021), Alzheimer’s disease (AD), and other prion-like neurodegenerative disorders (Ransohoff and Perry, 2009; Alpaugh and Cicchetti, 2021; Annadurai et al., 2021; Contiliani et al., 2021; Ritchie and Barria, 2021). Although transmissibility remains a unique characteristic of prion diseases, protein misfolding disorders share protein aggregation as a common mechanism as the disease spreads from cell to cell (Diack et al., 2016; Scheckel and Aguzzi, 2018).

Alzheimer’s and Prion’s pathologies share synaptic dysfunctions and axonal trafficking defects (Senatore et al., 2013; Zamponi et al., 2017; Soto and Pritzkow, 2018; Song et al., 2021) and similar alterations in the processing of neuronal membrane proteins, together with insoluble deposits of amyloid-β (Aβ) peptide and amyloid plaques. Because of the predictable course of the pathology along with anatomical locations (Braak and Braak, 1991; Scott et al., 1992; DeArmond, 1993; Eikelenboom et al., 1994, 2002; Zamponi and Pigino, 2019), prion disease in the murine model has been proposed as an important tool for experimental studies searching for mechanisms underlying chronic neurodegeneration (Betmouni et al., 1996; Diack et al., 2016).

Prions are proteinaceous infectious pathogens, devoid of functional nucleic acids that cause a group of fatal neurodegenerative diseases by self-propagating misfolding protein deposition and an associated inflammatory response (Carlson and Prusiner, 2021; Orge et al., 2021). Also known as transmissible spongiform encephalopathies, they can produce diseases in several species of mammals, such as Creutzfeldt-Jacob Disease in humans, scrapie in sheep, and bovine spongiform encephalopathy (Prusiner, 1996; Ayers et al., 2020). Prion agents are composed exclusively of a modified form of normal cellular prion protein (PrPC), which is then converted into an insoluble form resistant to the action of proteases (PrPSc) (Bolton et al., 1982; Prusiner, 1982; Carroll and Chesebro, 2019). This altered protein is deposited in the parenchyma of the central nervous system where it induces a chronic neuroinflammatory response (Betmouni et al., 1996; Carroll and Chesebro, 2019). Immunohistochemical studies have shown that PrP is the main component of the Aβ plaques in mammalian prion diseases (DeArmond et al., 1985; Priola, 2017). The experimental prototypic murine model of prion disease is well established and is generated by injecting the prion agent ME7 into the hippocampus of the inbred C57BL/6J mouse strain (Betmouni et al., 1996). Distinct mouse strains may show diverse incubation periods and end-stage neuropathological features (Borner et al., 2011). However, similar early synaptic loss precedes neuronal degeneration and associates with early behavioral deficits in distinct prion disease strains (Bruce et al., 1991; Cunningham et al., 2005a; Borner et al., 2011; Hilton et al., 2013). An extended incubation period, together with astrocyte and microglia activation, neuronal death, and neuropil vacuolization are typical neuropathological features of the mouse prion disease models (Williams et al., 1994; Betmouni et al., 1996). While tau phosphorylation changes are limited to the end-stage prion pathology (Asuni et al., 2010), induction of type I interferons (IFN-I) results in significant phenotypic alterations in microglia that accelerates disease progression (Nazmi et al., 2019). Neuronal loss develops late in the disease and occurs topographically through neuroanatomical pathways that vary according to the prion agent ‘strain’ and the animal model that is used (Fraser et al., 1989; Jeffrey et al., 2000; Reis et al., 2015). Heparan sulfate proteoglycan is associated with Aβ plaques (McBride et al., 1998), and neuronal loss seems to be associated with oxidative stress (Brown, 2005; Bettinger and Ghaemmaghami, 2020) and apoptotic mechanisms via the proteolytic activation of the protein kinase Cδ (Harischandra et al., 2014).

The mechanisms underlying prion-induced neurodegeneration have been widely investigated (Hughes and Halliday, 2017). Most of these studies point to the fact that the PrPC protein has important roles as an antioxidant molecule and an apoptotic regulator, and that its depletion in the course of the disease can induce direct neurotoxic effects by oxidative stress (Collinge, 2001; Shah et al., 2018). Recently, it has been demonstrated that chronic neuroinflammation, shared by many neurodegenerative disorders (Amor et al., 2014; Obst et al., 2017), is generated through the dysregulation of the NLRP3 inflammasome, a central component of the innate immune system that induces pro-inflammatory cytokine production and cell death (Coll et al., 2016; Holbrook et al., 2021).

### Social Isolation and Behavioral Changes in Chronic Neurodegenerative Diseases

The forced and prolonged social isolation caused by the COVID-19 pandemic has aggravated the psychiatric symptoms of older people with cognitive impairments (Barguilla et al., 2020; Manca et al., 2020). In fact, demented patients worsened in their cognitive, behavioral, and psychological symptoms, and the mortality rate associated with SARS-Cov-2 infection among these patients is very high (Toniolo et al., 2021b). The detrimental effects of social isolation on human health and cognition have been highlighted previously (House, 2001; Friedler et al., 2015). Despite these warning signs, there is a huge growth in the number of people who still live alone (Snell, 2017).

Evidence from both animal models and humans demonstrated the physiological benefits of social interaction (Krueger et al., 2009; Andrew and Rockwood, 2010; Karelina and DeVries, 2011; Holt-Lunstad, 2018). Therefore, detrimental effects of social isolation have been recognized systematically as a source of chronic stress associated with the increased prevalence of vascular and neurological diseases (Friedler et al., 2015). In addition, it has been suggested that reduction of social engagement between midlife and late-life periods can be predictive of functional disabilities (Guo et al., 2020), cognitive decline (Huang et al., 2020), and dementia and mortality (House et al., 1988; Saczynski et al., 2006; Daffner, 2010; Krivanek et al., 2021). Social isolation also increases the risk of chronic neurodegenerative diseases (Heneka and O’Banion, 2007; Amieva et al., 2010; Heneka et al., 2010; Lyman et al., 2013; Hajek et al., 2021) with differential neuro-immune markers for social engagement and loneliness (Walker et al., 2019). Previous findings in a mouse model of prion disease identified early behavioral and neuropathological changes associated with the inbred (C57Bl6J), as compared to the outbred (albino Swiss mouse) model of prion disease (Cunningham et al., 2005a; Borner et al., 2011). Nevertheless, little is known about the influence of social isolation on the progression of such diseases.

Previous studies using environmental manipulations in the triple transgenic mouse model of AD (3xTg-AD) were effective in modifying several behaviors but did not change genetically determined AD-like symptoms (Pietropaolo et al., 2009).

### Sedentary Life and Chronic Neurodegenerative Diseases

Environmental enrichment (EE) and physical exercise have been used to mimic an active lifestyle in humans and previous findings demonstrated that an active life slows AD progression (Silveira et al., 2018; de Freitas et al., 2020) and Huntington’s disease progression (van Dellen et al., 2000; Hockly et al., 2002; Spires et al., 2004), and extends the disease time course in experimental models. These animal models include the transgenic mice co-expressing familial AD-linked mutations on the amyloid precursor protein (APP) and presenilin 1 (PS1) (Lazarov et al., 2005), 1-methyl-4-phenyl-1,2,3,6-tetrahydropyridine (MPTP) and 6-hydroxydopamine (6-OHDA) Parkinson’s disease model (Faherty et al., 2005; Jadavji et al., 2006), and the mice expressing the human SOD1(G93A) gene mutation, the most common model of amyotrophic lateral sclerosis (Stam et al., 2008) and in the mouse prion disease (Bento-Torres et al., 2017).

Because EE and exercise moderate immune responses (Burtscher et al., 2021; Chastin et al., 2021; do Brito Valente et al., 2021; Filgueira et al., 2021; Proschinger et al., 2021; Sellami et al., 2021), and aging dysregulates immune responses (Brites, 2015; López-Ortiz et al., 2021; Martinez et al., 2021; Mathot et al., 2021), we previously hypothesized that EE and aging would, respectively, delay and accelerate prion disease progression (Bento-Torres et al., 2017). However, we found that after intracerebral injection of the ME7 agent into the dorsal striatum, aged mice exhibited significantly reduced disease progression when compared to young mice injected with ME7 (Bento-Torres et al., 2017).

To illustrate the effects of exercise and EE on disease progression after intraperitoneal injection, we selected two hippocampal-dependent tasks: burrowing (Deacon et al., 2001; Deacon, 2009) and the Morris water maze (Morris et al., 1982; Morris, 1984). Burrowing behavior was found to be the most sensitive task to detect early hippocampal dysfunction in mouse prion disease, which coincided with the onset stage (Deacon et al., 2001; Cunningham, 2005). Similarly, the Morris water maze task in rat AD models was found to detect subtle impairments in aged mice (Sun et al., 2019).

A systematic review dedicated to the identification of the beneficial effects of physical exercise in AD suggests that aerobic exercises are an effective intervention that can attenuate the neuropsychiatric symptoms as the disease progresses (Mendonça et al., 2021). Evidence also indicates that physical exercise leads to the release of-induced myokines, a group of peptides produced and secreted by skeletal muscles during exercise (Pedersen, 2009), which have been shown to haveneuroprotective roles (Petersen and Pedersen, 2005; Astrom et al., 2010; de Freitas et al., 2020; Lee B. et al., 2021). Similarly, EE seems to prevent microglia-mediated neuroinflammation (Almutairi et al., 2016).

### Exacerbated Inflammatory Response and Sedentary Lifestyle

Chronically activated neuroinflammatory processes in neurodegenerative diseases play a central role in their pathogenesis (Heneka et al., 2015; Ransohoff, 2016). Because microglial proliferation is a major component in the progression of chronic neurodegeneration (Gómez-Nicola et al., 2013; Subhramanyam et al., 2019; Azam et al., 2021) and the microglial innate immune response in prion disease (Peggion et al., 2020) is also considered to contribute to the activation of the peripheral immune system at draining lymph nodes and the spleen (Vincenti et al., 2015), it is thought that interactions with other immune cell populations may accelerate the spread of neurodegeneration in prion disease brain (Mabbott et al., 2020). Indeed, splenectomy before intraperitoneal prion infection was shown to extend survival times but had no effect on disease pathogenesis when intracerebral injections of prions were performed (Fraser and Dickinson, 1970; Mabbott et al., 2020). Following peripheral exposure, many prions replicate in the lymphoid tissues before entering the central nervous system, and prion pathogenesis is impaired dramatically in aged mice when compared with young animals (Brown and Mabbott, 2014). Thus, owing to the compromised immunosenescence microglial response in aged mice (Brites, 2015; Carvalho-Paulo et al., 2021), a stronger inflammatory response would be expected in young mice (Bento-Torres et al., 2017).

Previous findings in the triple transgenic mouse model of AD, which develops both Aβ plaques and neurofibrillary tangles mimicking the temporal- and regional-specific profile of the human disease, suggested that impairment of the peripheral immune system and neuroimmune communication contribute to premature aging of these mice (Giménez-Llort et al., 2012). Similar cross-talk between peripheral immune cells and microglia has been described in AD and these peripheral immune cells may help in Aβ peptide clearance and modulation of microglia response (Dionisio-Santos et al., 2019). In addition, chronic neuroinflammation in normal aging (Groh et al., 2021) and age-related chronic neurodegenerative diseases, such as AD (Gate et al., 2020) and Parkinson’s disease (Galiano-Landeira et al., 2020), have been found to include innate and adaptive immune cell dysfunction (Carrasco et al., 2021; Lutshumba et al., 2021).

Thus, the intense microglial activation in chronic neurodegenerative diseases, under influence of both peripheral and central homeostatic changes, damages healthy neural tissue, and then, in response to the factors secreted by dead or dying neurons, microglial activation is chronically maintained and the associated neuroinflammation leads to progressive self-propagating damage (Xu et al., 2016; Subhramanyam et al., 2019).

Microglial activation and neuroinflammation have been shown to be modulated by voluntary exercise and EE (Duggan and Parikh, 2021), which can slow down disease progression. Indeed, we have previously demonstrated that EE and exercise in a dose-dependent way can attenuate neuroinflammation in the ME7 mouse model of prion disease (Bento-Torres et al., 2017). It has been also described that the microglial response in the 3xTg-AD mouse model is differentially modulated by voluntary wheel running and enriched environments, as evidenced by the presence of hypertrophic microglia (increased surface, volume, and somata volume) in the standard environment of laboratory cages, as compared with mice preserved in enriched cages (Rodríguez et al., 2015).

Previous consensus established that oxidative stress, DNA damage, mitochondrial dysfunction, excessive accumulation of misfolded proteins, synaptic impairment, and damage to microRNA (miRNA) processing and inflammation (Brites, 2015; Lutshumba et al., 2021) maybe associated with age-related changes in microglia (Koellhoffer et al., 2017; Costa et al., 2021; Triviño and von Bernhardi, 2021). Indeed, the immunosenescent phenotype of microglia is marked by dystrophic morphology, elevated expression of inflammatory markers, reduction in the release of neuroprotective factors, alterations in the transcriptomic profile and phagocytic activity, together with modifications in their secretome cargo (Niraula et al., 2017; Angelova and Brown, 2019; Greenwood and Brown, 2021). These alterations may explain the reduction of morphological changes in the aged ME7 prion-infected mice (Bento-Torres et al., 2017).

Astrocytes can also change their homeostatic phenotypes in response to acute and chronic pathologies, showing reactive subtypes with increased expression of the glial fibrillary acidic protein (GFAP) (Anderson et al., 2014). In the ME7 prion disease mouse model, the analysis of the hippocampal proteome revealed a predominantly activated astrocyte signature (Asuni et al., 2014).

Astrocyte reactivity in the ME7 prion disease mouse model is influenced by EE and exercise, which decreases neuroinflammation and cell reactivity (Bento-Torres et al., 2017). This is also true for AD models (Kelly, 2018). In fact, the enriched environment and physical exercise have been widely used in experimental models of chronic neurodegenerative diseases to slow the progression and to investigate the mechanisms underlying this protection (Rodríguez et al., 2011; Do et al., 2018; Kim et al., 2019; Pena et al., 2020). Exercise on the treadmill for 5 days per week reduced disease progression in the 3xTg-AD mice, which was associated with lower Aβ plaque burden and neuroinflammation, and improved mitochondrial function and neurogenesis (Kim et al., 2019). Similarly, beneficial effects were described after regular resistant training in 3xTg-AD mice with reduction of the Aβ peptide in the hippocampus and increased concentration of insulin-like growth factor 1 (IGF-1) (Pena et al., 2020). Although less explored, the Huntington’s disease mouse model R6/1HD submitted to voluntary exercise, using running wheels and subsequently enriched environment, seemed to synergistically increase hippocampal neurogenesis with old adult-generated neurons, microglia, and astrocytes, without revealing mutant huntingtin immune reactive aggregates (Ransome and Hannan, 2013).

Astrocyte reactivity by upregulation of the glial fibrillary acidic protein astrocyte reactivity in chronic neurodegenerative diseases is associated with nuclear factor kappa B (NF-κB) activation and remodeling of chromatin with subsequent transcription of proinflammatory genes (Villarreal et al., 2021). Sustained inflammatory signaling by activated microglia in to astrocytes and the established crosstalk known to exist between microglia and astrocytes induce astroglial pathological remodeling and the exacerbation of neuronal death (Jha et al., 2019; Verkhratsky et al., 2019; Matejuk and Ransohoff, 2020).

### Infection and Chronic Neurodegeneration

Among the infectious diseases, there has been emerging evidence that infectious agents can be part of the environmental risk factors for the aggravation of neurological disorders (Toniolo et al., 2021a; Wouk et al., 2021). This is the case of chronic neurodegenerative disorders, such as AD (Itzhaki and Wozniak, 2010; Giridharan et al., 2019; Lopez-Rodriguez et al., 2021; Mathis et al., 2021), Parkinson’s (Munoz-Pinto et al., 2021; Rosen et al., 2021), and experimental prion diseases (Lins et al., 2016; Nazmi et al., 2019). Pre-existent inflammatory conditions, such as those associated with chronic neurodegenerative diseases in humans and mice, seem to be aggravated by both peripheral and central infections (Combrinck et al., 2002; Cunningham et al., 2005b; Holmes and Butchart, 2011; Naughton et al., 2020; Zhou et al., 2021). Indeed, cognitive deficits of patients with AD are further increased after a systemic infection, and this is preceded by an increase in the release of interleukin-1β (Holmes and Butchart, 2011). In addition, mouse prion disease shows more intense neuropathological features and faster disease progression after systemic and central endotoxin challenges (Combrinck et al., 2002; Cunningham et al., 2005b; Hennessy et al., 2015, 2017; Lins et al., 2016; Nazmi et al., 2019).

Previous findings using an intranasal Piry neurotropic virus infection, intrahippocampal injection of ME7 prion strain, or normal brain injection, demonstrated that virus-infected prion-diseased mice exhibited higher microglial morphological reactivity and more severe behavioral outcomes than ME7 prion-diseased mice not infected with virus (Lins et al., 2016). Although virus infection *per se* did not change the number of microglia in CA1, virus infection in prion-diseased mice (at 17 weeks post-injection) induced changes in the number and morphology of microglia. We suggested that virus infection exacerbated microglial inflammatory response in prion-infected mice, thus aggravating chronic neurodegeneration (Lins et al., 2016).

SARS-CoV-2 has been found to invade the brain via the olfactory, gustatory, and trigeminal pathways, especially at the early stage of infection (Liu J. M. et al., 2021). Its neuroinvasion route through nasal epithelium (Yachou et al., 2020) is similar to that of many other RNA viruses (Freitas et al., 2020; Awogbindin et al., 2021), including the Piry arbovirus used to infect the mouse prion disease model (de Sousa et al., 2015). We found that the Piry virus interaction with ME7-associated chronic neurodegeneration induces progressive exacerbation of microglia and astrocyte morphological alterations. These findings demand further exploration and discussion of the potential mechanisms by which microglia and astrocyte dysregulated responses (Murta et al., 2020) may contribute to post-COVID-19 neurological sequelae (Mishra and Banerjea, 2020) that are associated with the aggravation of chronic neurodegenerative diseases (Sita et al., 2021).

Neuropathological examination of many areas of the central nervous system in aged patients infected with SARS-CoV-2 who died during the disease revealed signs of neuroinflammation with astrogliosis and microglial activation. Microglial nodules and neuronophagia, most prominent in the brainstem, with hypoxic/ischemic changes in many areas of all examined brains, were also evident (Matschke et al., 2020; Thakur et al., 2021). In this study, it is important to highlight that 44% of the elderly patients also revealed neuropathological signs of ongoing neurodegenerative diseases (Thakur et al., 2021).

Following SARS-CoV-2 respiratory infection, choroid plexus epithelial cells are affected by signals from peripheral inflammation followed by activation of the immune system of the brain, such as differential expression of microglial and astrocytic inflammatory-associated genes, dysregulated homeostasis, and peripheral T-cell neuroinvasion (Schwabenland et al., 2021; Yang et al., 2021). These studies showed no molecular traces of SARS-CoV-2 in the brain, but broad cellular perturbations of the choroid plexus leading to the spread of peripheral inflammation mediators into the brain. These findings suggest that the severity of the neuropathological changes is not caused by direct infection of the virus in the brain parenchyma, but rather from systemic inflammation. Thus, it remains open the possibility that similar pathological changes in patients who survived from COVID-19 may aggravate ongoing chronic neurodegenerative diseases.

It has been noted that elderly patients infected with COVID-19, who had episodes of delirium, showed significant hyperactivation of microglia in the hippocampus. Together with the inflammatory lesions of the brainstem and the presence of topographic signs and symptoms, in the absence of specific signs of encephalitis associated with SARS-CoV-2, such features constitute the so-called COVID-19 encephalopathic syndrome (Poloni et al., 2021). While delirium in humans and sickness behavior in experimental models are transient, there is compelling evidence that such systemic immune responses and inflammation give rise to long-lasting consequences for the brain, particularly in aged individuals (Lutshumba et al., 2021). This condition of long-lasting symptoms experienced by many patients who have suffered from acute COVID infectious is now referred to as the long COVID syndrome (Hugon et al., 2021; Taribagil et al., 2021).

It is, therefore, reasonable to infer that a patient who has survived from COVID-19 encephalopathic syndrome, experiencing or not experiencing long-COVID symptoms, may suffer exacerbated neuroinflammation that will accelerate/aggravate the progression of pre-existing chronic neurodegenerative disease.

It is important to highlight, however, that although pathogenic mechanisms of age-related neurodegenerative disorders include the seeded aggregation of disease-specific proteins, as in the prion disease model (Walker and Jucker, 2015), the incomplete similarity of events observed in these diseases does require a cautionary approach to the generalized use of prion disease as a proxy for immune response investigation in all prion-like disorders (Guest et al., 2011). In addition, the possibility of differential mechanisms by which peripheral or central infections interact and aggravate abnormal disease-specific protein aggregation and damage to the brain tissue remains to be investigated in detail in each of those diseases. Finally, it is also imperative to investigate if exogenous and endogenous risk factors for each disorder interact with infections, and how this interaction contributes to misfold and progressive accumulation of protein clumps. It is expected that future studies may reveal new opportunities for therapeutics and also for new public health risk identification (Cashman, 2015).

### Chronic Neurodegeneration, Virus Infection, and miRNAs

miRNAs can regulate innate and adaptive immunity by regulating microglia activation, astrocyte reactivity, and by controlling the egress of peripheral immune cells, such as neutrophils, macrophages, T cells, and B cells (Gaudet et al., 2018). miRNAs play an emerging and important role in the interplay between viruses and host cells (Liu W. et al., 2021; Pandey et al., 2021), and potential interaction between SARS-CoV-2 and human miRNAs have been predicted and tested (Marchi et al., 2021; Siniscalchi et al., 2021). Neurodegenerative diseases, such as AD, Parkinson’s disease, Huntington’s disease, multiple sclerosis, and prion-like diseases, are characterized by the deposition of misfolded proteins, such as Aβ, tau, α-synuclein, huntingtin, and prion proteins (Khan et al., 2021). Deregulated miRNA profiles are associated with the development and progression of AD. They are known to induce the activation of microglia into disease-associated polarized phenotypes that aggravate neurodegeneration. However, the modulation of the inflammatory-associated miRNAs may also encourage microglia to engage in reparative mechanisms (Fernandes et al., 2018; Brites, 2020). The communication between microglia and astrocytes is mediated through exosomes, which are small extracellular vesicles, and by soluble factors as cytokines. Exosomes are enriched in lipids, proteins, and genetic material, and their cargo in miRNAs was shown to have an important effect on the behavior of recipient cells. Dysregulated production of miRNA has been reported to cause neuroimmune dysfunction (Yang and Zhu, 2019) and encourage neurodegenerative processes in AD mouse models and patients (Guedes et al., 2014; Brites, 2020; Kim et al., 2020). It has been proposed that the SARS-CoV-2 gene product Spike is able to modify the host exosomal cargo, thus, facilitating its transportation to distant uninfected tissues and organs initiating a severe inflammatory cascade (Mishra and Banerjea, 2021). Spike transfected cells release a significant number of exosomes enriched in miRNA(miR)-148a and miR-590 that are internalized by microglia and are able to upregulate the proinflammatory gene expression, such as tumor necrosis factor alfa (TNF-α) and interferon beta (IFN-β), which can promote the unwanted exacerbation of inflammatory microglia responses (Mishra and Banerjea, 2021).

## Concluding Remarks

Social isolation, sedentary life, and infection are all associated with the restrictions imposed by the COVID-19 pandemic rules and the presence of the virus. In this study, we have revisited the effects of sedentary life and infections on mouse prion disease progression, as a proxy for the exacerbated immune response of prion-like chronic ongoing neurodegenerative diseases. Our previous study with mouse prion disease has demonstrated that these influences contribute to the undesirable aggravation of astrocyte reactivity and microglial activation, which results in more severe behavioral outcomes, and acceleration of disease progression. We anticipate that the SARSCoV-2 infection may similarly potentiate ongoing chronic neurodegenerative disease progression in patients surviving to COVID-19. Our findings, and those of other researchers, have demonstrated the benefits of EE and physical exercise, while emphasizing that an active lifestyle may reduce neuroinflammation, cognitive decline, and behavioral abnormalities and may slow disease progression. Thus, a more physically active lifestyle might also be expected to positively impact on the downstream sequelae associated with SARS-CoV-2 infection.

## Author Contributions

All authors contributed substantially to the conception or design of the study; the acquisition, analysis, or interpretation of data for the study; drafting the study or revising it critically for important intellectual content; or final approval of the version to be published; and agreed to be accountable for all aspects of the study in ensuring that questions related to the accuracy or integrity of any part of the study are appropriately investigated and resolved. CWPD, DB, and DA participated in the data interpretation and writing of the final version.

## Conflict of Interest

The authors declare that the research was conducted in the absence of any commercial or financial relationships that could be construed as a potential conflict of interest.

## Publisher’s Note

All claims expressed in this article are solely those of the authors and do not necessarily represent those of their affiliated organizations, or those of the publisher, the editors and the reviewers. Any product that may be evaluated in this article, or claim that may be made by its manufacturer, is not guaranteed or endorsed by the publisher.
